# Can Seeding in the Clinic Reach a Wide Audience? A Proof of Concept Study on Spreading a Health Message About Juvenile Idiopathic Arthritis Using a Shareable Online Video

**DOI:** 10.2196/ijmr.4608

**Published:** 2016-02-22

**Authors:** Michaela Fay, Tim Rapley, Helen Foster, Clare Pain, Craig Gerrand

**Affiliations:** ^1^ Institute of Health and Society Newcastle University Newcastle Upon Tyne United Kingdom; ^2^ Institute of Cellular Medicine Newcastle University Newcastle Upon Tyne United Kingdom; ^3^ Department of Paediatric Rheumatology Alder Hey Children's NHS Foundation Trust Liverpool United Kingdom; ^4^ Department of Orthopaedics Newcastle Upon Tyne Hospitals NHS Foundation Trust Newcastle Upon Tyne United Kingdom

**Keywords:** juvenile arthritis, video-audio media, patient education, Internet

## Abstract

**Background:**

Shareable online video offers the potential for spreading a health message across online and real world social networks. Seeding a message in a clinical setting may be advantageous.

**Objective:**

To investigate the potential of an online video to spread a health message about juvenile idiopathic arthritis (JIA) when delivered or seeded in a clinical setting and investigate factors that influence sharing behavior.

**Methods:**

Multimethod proof of concept study. Concepts for two different styles of video were developed using focus groups and interviews and reviewed by an online market research panel. We compared dissemination of the two videos from two specialist pediatric rheumatology clinics in NHS Hospitals. Participants were 15 patients, family members, and clinical staff with knowledge of JIA at concept stage; 300 market research panel members in development stage; and 38 patients and their parents or guardians in the seeding stage. Newly diagnosed patients with JIA and/or parents or guardians were invited to view and share an online video with a health message about JIA across real-life and electronic social networks. Main outcome measures were viewing statistics, sharing behavior and patterns, and participant feedback.

**Results:**

Of 38 patients and/or their parents or guardians given links, 26 visited the video webpage and shared the link, 2 visited and did not share, and 10 did not visit. Most links were viewed and shared within a few days. A total of 3314 pageviews were recorded with a mean of 89.6 pageviews per link (range 0-1245). Links were accessed from 26 countries, with most viewers in the United Kingdom (82.5%). Mothers were the most active group of sharers.

**Conclusions:**

Distribution of a video link in a clinical setting may be an effective way to spread a health message. Parents or guardians of children with JIA are more likely to share a link than young people. Dissemination depends on a small number of active sharers, the content of the video, and the willingness of participants to share health information about themselves.

**Trial Registration:**

UK Clinical Research Network Study Portfolio ID (UKCRN): 13747; http://public.ukcrn.org.uk/Search/StudyDetail.aspx?StudyID=13747 (Archived by WebCite at http://www.webcitation.org/6eeXlMmM6).

## Introduction

Health promotion and early diagnosis are core components of the National Health Service Improving Quality program [1]. Traditional multiplatform awareness campaigns, however, can be resource intensive with a finite lifespan, and their impact and cost effectiveness may be difficult to measure [2]. In contrast, the sharing of online content may disseminate health messages at relatively low cost. The potential reach of online messages is increasing: 73% of the UK population use the Internet and of these, 71% use it to gather health information [3]. One in 20 Google searches is for health information [4]. Furthermore, improving the use of digital technologies for health is a priority for the National Health Service [5].

Viral campaigns can reach large numbers of people through active sharing, but success depends upon the willingness of individuals to share messages. Shared online health messages are not simply passed from producer to consumer but are mediated before dissemination across social and other networks (eg, email). The potential advantages of online sharing of health messages include cost-effective dissemination of bottom-up advice, greater reach than read-only information, the ability to trigger debate and generate support within networks, and encouragement for patients to “come out” as living with a chronic condition. Open discussion within social media forums can decrease stigma attached to health conditions such as mental illness [6].

We were therefore interested in finding out whether seeding a viral campaign in a clinical setting could be effective in spreading a health message. We thought this could be successful because the message came from a trusted source, and members of patients’ social networks might be interested in the patients and their new diagnoses. However, we recognized this approach could fail if there was reluctance to disclose personal information (ie, participants felt vulnerable) [3] or if there was stigma associated with the condition. Furthermore, successful dissemination online depends on the tone and content: a humorous message about sexually transmitted diseases may be more widely shared than a serious one [7].

The diagnosis of musculoskeletal conditions such as juvenile idiopathic arthritis (JIA), muscular dystrophies, and bone cancer in children and young people is often delayed, which has a negative impact on clinical outcomes and experience of care [8-11]. We therefore identified JIA as an appropriate condition for this study, recognizing the approach may work for other conditions as well.

We aimed to explore in this proof of concept study whether a health message with a shareable online video would be disseminated after initial distribution in a clinical setting by newly diagnosed children and young people with JIA and their parents or guardians. We also aimed to evaluate the feasibility of this approach and factors that might influence the distribution of such a video.

The specific objectives were

- To investigate whether an online video distributed in a clinical setting is shared
- To determine whether video style and content influence sharing
- To obtain user feedback in order to develop this approach

## Methods

**Figure 1:**
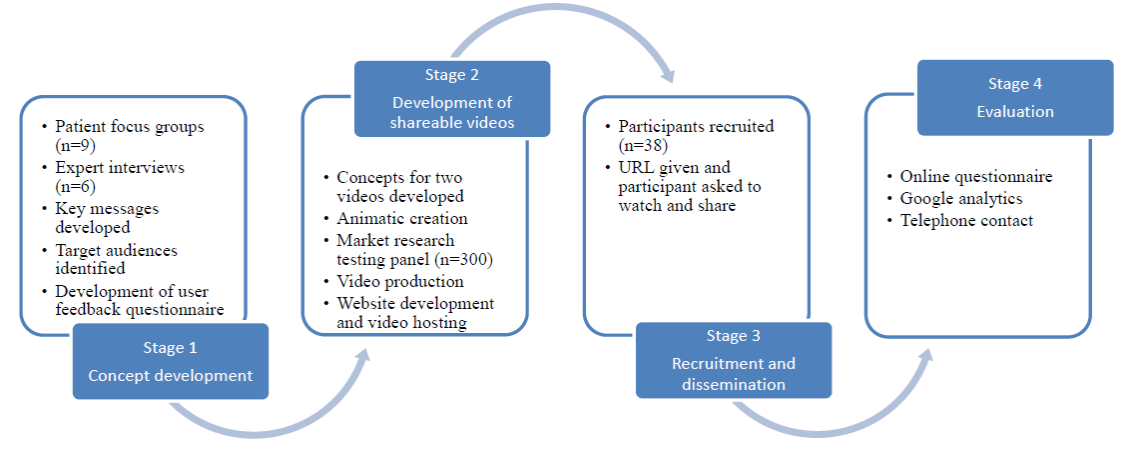
Project overview.

This was a multimethod study in 4 stages (see Figure 1). Ethical approval was obtained from the Local Regional Ethics Committee. Informed consent from parents or guardians and assent from children were obtained as appropriate and all data were anonymized before analysis.

### Concept Development

In this first stage we explored ideas about online sharing and developed key concepts for video development. Focus groups were held with newly diagnosed and established children and young people with JIA (2-16 years of age) and their parents or guardians. Interviews were held with other interested parties including health care professionals caring for patients with JIA. Participants were recruited from one center (Newcastle).

A researcher (MF) led each focus group using a topic guide including the following themes: (1) awareness of JIA before diagnosis, (2) journeys to diagnosis and information seeking, (3) views about information that others should have and how to communicate that information, (4) how children and young people and parents or guardians share experiences, and (5) the reactions of others to a diagnosis of JIA. In interviews with other interested parties, themes emerging from the focus groups were further explored. Focus groups and interviews were audio recorded, and key themes were identified using grounded theory [12].

### Video Development

In the second stage, a digital communications agency [13] developed 2 contrasting videos using findings from Stage 1. This process included script development, refinement, animatic (draft video storyboards) creation, market research testing, casting, filming, and editing.

To ensure videos were appropriate, informative, and likely to be shared, animatics were reviewed by an independent market research testing panel comprising 300 people without previous first-hand experience with JIA. Panel members were grouped for analysis as follows: 13- to 16-year-olds (male or female without children), 17- to 25-year-olds (male or female, including parents or guardians), and mothers of children up to 16 years of age. Panel feedback was incorporated into the final scripts. Two videos, each approximately 1 minute in duration, were cast, filmed, and edited to agreed final versions.

### Recruitment and Video Dissemination

Participants were recruited using a criterion sampling method in which children and young people within 6 months of diagnosis of JIA were identified in 2 centers (Newcastle upon Tyne and Liverpool). The parent or guardian was sent an information sheet before the clinic appointment, and participants (patients or their parents/guardians) were recruited in the clinic by the researcher or a clinical staff member.

Participants were handed a postcard with a unique web link (bit.ly) and asked to access the link, view the video, and share it across their real-life and electronic social networks. Each link was associated with one of the 2 randomly assigned videos. Researchers were blinded to the allocation. Within 4 weeks, participants were interviewed by telephone for feedback about the study.

Videos were hosted on a private, purpose-built website comprising multiple pages with unique 3-digit identifiers, each of which could be tracked. Each page comprised a video and share buttons (Facebook, Twitter, LinkedIn, Google+, Blogger, Reddit, Tumblr, and email). At the end, viewers were asked to complete an online questionnaire (SurveyMonkey) including demographic details, opinion of the video, reasons for sharing or not sharing, and social network use.

### Evaluation

Standard web analytic tools (Google Analytics) tracked link activity. Google Analytic algorithms count a pageview when a user loads a page. Technology platform and some other metrics are reported by session: a session starts when a user accesses a website and ends after 30 minutes of inactivity or when the user moves to another website. Data collection was terminated when activity fell to very low levels, approximately 6 weeks after recruitment of the final patient. Analysis included geographic location of viewers, viewing platform (eg, mobile or tablet), referring site, number of pageviews and sessions, and time spent on the website. Results for the 2 videos were compared. Participants were telephoned 2 weeks after recruitment. Results from the online questionnaire were compiled using standard metrics.

## Results

### Concept Development

Two focus groups were held, each comprising children and young people with JIA and their parents or guardians (n=9). Participants felt there was little awareness among the public and health care professionals that arthritis can affect children and treatment is often successful. Because effective treatments are available, appropriately treated JIA is an invisible albeit chronic condition. Although this means children and young people may not have to disclose their diagnosis, low visibility of physical changes may perpetuate lack of awareness and therefore delayed diagnosis. Interestingly, participants were concerned the public may have little interest in JIA because it is a manageable condition.

Participants felt that children and young people and parents or guardians are often better informed than health professionals and indeed may have suggested the possibility of JIA to their general practitioner before diagnosis. They also reported low levels of awareness in schools that arthritis affects children and young people. Therefore, support is often lacking, affected families are left to inform schools, and children and young people have to negotiate with peers. Adolescents may be reluctant to tell others about their condition to avoid being perceived as “boring” or “crippled” if unable to participate in certain activities, and this may encourage teasing or bullying.

Although fathers frequently attend hospital appointments with their child, participants felt mothers would be more engaged in information gathering, disease management, and exchange with others (eg, extended family and school). Participants identified 2 key target audiences: mothers of children and young people with arthritis and mothers of adolescents and young adults with arthritis. Videos provoking an emotional reaction were thought likely to be shared more widely than those resembling a fact sheet.

Other interested parties (n=6) were interviewed: a general practitioner with a special interest in pediatric rheumatology, a clinical nurse specialist in pediatric rheumatology, 2 consultant pediatric rheumatologists, the founder of a large parent or guardian support and awareness-raising network, and the communications officer of a relevant medical charity. Additional issues that emerged were around video style and content. Participants felt that to be widely shared, videos should be cute or humorous with key messages that children and young people can have arthritis and with timely treatment many maintain good quality of life. In addition, videos should not be heavily educational or overly alarmist while discussing symptoms (eg, persistent swelling, pain, stiffness). Participants suggested the most motivated sharers would be mothers of younger children, parents or guardians of children with more severe disease, and those who had suboptimal diagnostic experiences.

### Video Development

Concepts and animatics for 2 contrasting videos were developed by the creative team. The 2 target audiences in Stage 1 were selected: parents or guardians (mothers in particular) of younger children and adolescents and young adults. Key messages were

- Anyone can get arthritis, at any age
- Early treatment is important

The video concepts were (1) “How old do you need to be…?” (Multimedia Appendix 1) and (2) “Old to young” (Multimedia Appendix 2).

**Video 1 concept: “How old do you need to be...?”**

A series of children approximately 4-10 years of age are shown answering “How old do you need to be...?” questions: “How old do you need to be to make a cup of tea/drive a bus/buy a house/get married/bake a cake/go on an airplane by yourself?” The answers, delivered to camera, are diverse, lighthearted, humorous, and real. Eventually we arrive at the last question—“How old do you need to be to get arthritis?”—to which children reply with high numbers (72, 65, etc). The last child we see tells us you can be any age to get arthritis. It ends with the caption “You don't have to be old to get arthritis” and a call to action to share the link.

**Video 2 concept: Old to young.**

An older woman is seen in a teenager's bedroom playing drum and bass on a turntable and moving to the music. Her voice is dubbed by a teenage female talking about the joy of listening to music and dancing. As her tone changes from happy and animated to sad, she talks about restricted movement, swelling, and pain, which at first she could not make sense of. The video culminates in a “big reveal” where the viewer discovers that the old woman is in fact a teenage female. The viewer sees the old woman put down her headphones, and the camera moves up to meet the face of the teenager whose voice we have been hearing. The message is that what started out as diffuse and inexplicable pains was, in fact, JIA. The video ends with the caption “You don’t have to be old to get arthritis” and a call to action to share the link.

Market research panel feedback about the animatics suggested the concepts successfully communicated key messages and would appeal to the target audiences. Concept 1 was considered more humorous and shareable and likely to appeal to mothers of young children. Concept 2 was considered more shocking and likely of greater appeal to 13- to 16-year-olds. Although the panel suggested 13- to 16-year-olds shared online video content most frequently, mothers were felt most likely to share a health message about their children online. Suggested barriers to sharing included lack of relevance, reluctance to share content about health, and self-consciousness about sharing content.

### Recruitment and Dissemination

A total of 38 participants were recruited between January and May 2014 in 2 centers (23 from Newcastle upon Tyne and 15 from Liverpool). Links were distributed to the participants and/or their parents or guardians: 21 were to Video 1 (“How old do you need to be…?”) and 17 to Video 2 (“Old to young”). One participant mislaid the paper link which was replaced. Only one potential participant, a 16-year-old male, declined. In general, even when offered to children and young people considered old enough to use them, the links were accepted by a parent, guardian, or other family member instead.

The mean age of the children and young people was 7.6 years (range 2-15); 28 were female and 8 male (2 unknown). Age, gender, and video allocation did not vary significantly by center. Children and young people allocated Video 1 were not significantly different from those allocated Video 2 in terms of age or gender (Video 1, mean age 7.5 years, 18/21 female; Video 2, mean age 8.6 years, 10/17 female; *P*=.47 and *P*=.14, respectively).

### Evaluation

#### Viewing, Sharing, and Pageviews

Of 38 distributed links, 10 were not accessed. The remaining 28 links achieved a total of 3236 pageviews (median 32 pageviews per link; range 2-1257).

**Figure 2:**
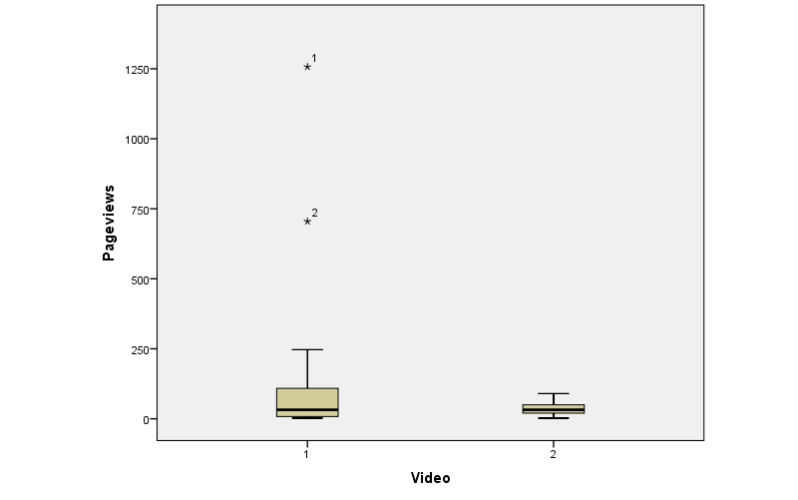
Box and whisker plot showing distribution of numbers of pageviews for each link grouped by the linked video.

Of 20 links to Video 1, 1 was not accessed. The remaining 19 links achieved a total of 2868 pageviews (median 32 pageviews per link; range 2-1257). One 12-year-old shared the link with her teacher and classmates but did not share online. Of 18 links to Video 2, 9 were not viewed. The remaining 9 links achieved a total of 368 pageviews (median 32 pageviews per link; range 2-90) (Figure 2). Visitors were recorded as *new* in 82.10% (2657/3236) of pageviews and *returning* in the remainder. The average session duration was 1:14 minutes.

**Figure 3:**
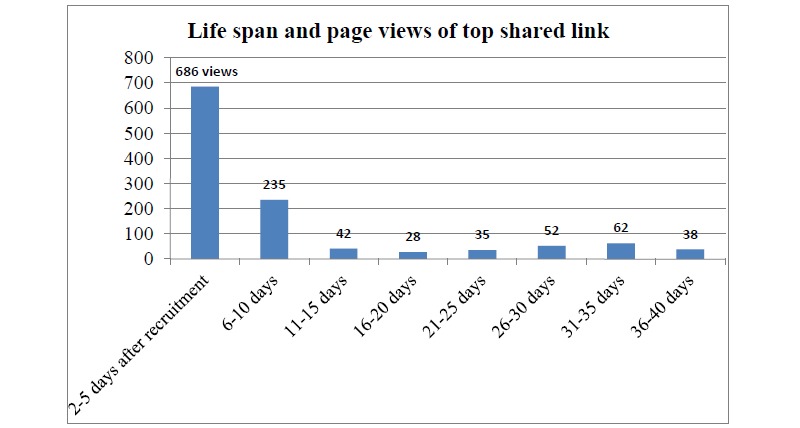
Life span and page views of top shared link.

Most links were viewed and shared within a few days. The lifespan of links was variable. For example, the most shared link was first shared 2 days after recruitment and generated 837 pageviews in the first week, decreasing in the subsequent 6 weeks (Figure 3). The second most shared link had a lifespan of approximately 3.5 weeks, with a peak of 469 pageviews on the second day followed by a decline in activity.

#### Geographic Spread

Links were accessed from 26 countries (Table 1). Most viewers were in the United Kingdom (82.72%) and the United States (9.05%). Within the United Kingdom, most were in the North East and London. The 2 most shared links had the greatest geographical reach at 11 and 13 countries.

#### Technology Platforms

Of 3236 pageviews, 2175 (67.21%) were from social networks, 624 (19.28%) from the original bit.ly links, and 437 (13.50%) from direct links. The social networks driving viewers were Facebook (2143/2175, 98.53%), Twitter (16/2175, 0.74%), Mums in the Know (14/2175, 0.64%), and Google+ (2/2175, 0.09%). Of the 3236 pageviews, 2417 (74.69%) were on mobile or tablet devices.

#### Online Questionnaire Feedback

There were responses to 78 online questionnaires. All respondents either had arthritis or had a child with arthritis. The majority were female (66/72, 92%) and aged 25 to 44 years (50/72, 69%). Most (48/60, 80%) had been through or were planning to complete higher education.

Of 67 respondents to this question, 16 (24%) received the link directly from the research team, 30 (45%) via social networking sites, 5 (7%) from friends and 3 (4%) from a family member; 23 (34%) received the link from someone with JIA or whose child had JIA.

Of 72 respondents reporting their social media use, 70 (97%) used Facebook, 28 (39%) Twitter, 17 (24%) Instagram, 14 (19%) Pinterest, 6 (8%) Google+, 3 (4%) Tumblr, 2 (3%) Reddit, and 1 respondent kept a personal blog; 34 (47%) reported being logged on most of the time and 33 (46%) at least daily.

Most respondents (60/77, 78%) shared the video (Table 2). The most popular reason for sharing was that the viewer or child had JIA and wanted others to know about it. Videos were most often shared on social networking sites (48/59, 81%) with friends (24/59, 41%) or family members (17/59, 29%) and less often with work colleagues (4/59, 7%).

**Table 1:** Geographic distribution of viewers.

Country/Territory	Pageviews n (%)
United Kingdom	2677 (82.72)
United States	293 (9.05)
Australia	92 (2.84)
Canada	40 (1.23)
Ireland	36 (1.11)
Singapore	12 (0.37)
Italy	10 (0.31)
Netherlands	10 (0.31)
New Zealand	10 (0.31)
Germany	6 (0.18)
Spain	6 (0.18)
France	6 (0.18)
Czech Republic	4 (0.12)
Lithuania	4 (0.12)
Thailand	4 (0.12)
South Africa	4 (0.12)
Others	22 (0.68)
Total	3236 (100)

**Table 2:** Reasons for sharing or not sharing the video.

Response		Respondents n (%)
**Shared** **(n=59)**		
	I/my child has JIA and I want others to know about it too	48 (81)
	I know someone who has arthritis	10 (17)
	As a favor to the person/website who sent me the link	8 (14)
	It was touching	5 (8)
	It was funny	4 (7)
	It was informative	3 (5)
	It was different	2 (3)
**Not shared** **(n=11)**		
	I don’t usually share things online	5 (46)
	I/my child has JIA, but I don’t want to shout it from the rooftops	3 (27)
	I didn’t like the look of it	3 (27)
	I don’t know anybody who would be interested	2 (18)
	I didn’t have time, and I forgot all about it	1 (9)

When asked what they liked about the video, responses were “It reminded me of myself/my child/someone I know who has got JIA” (31/65, 48%), “The way it talks about JIA” (24/65, 37%), “It was informative” (18/65, 28%), “It was cute” (15/65, 23%), “It made me feel quite emotional” (8/65, 12%), and “It was funny” (5/65, 8%). Comments included the following:

It plays on stereotypes and shows people their preconceptions are wrong.

It’s brief and to the point without being overly emotional. I don't share much about my daughter's JIA as it is personal to her but nor do I want her to feel ashamed or different—knowledge empowers and reduces prejudice. I was happy to share this.

Some respondents (17/77, 22%) reported viewing but *not* sharing the video (Table 2). The most frequent reason for not sharing was “I don’t usually share things online” (5/11, 46%). Comments included “My child is only 2 and . . . has stiff or sore joints . . . This wasn’t mentioned in the video,” the video was “not informative enough,” and “Young people are likely to turn off as soon as the elderly person appears.” Others reported it was irrelevant to their circumstances or, anticipating stigma or bullying, were not comfortable publicizing that their child had JIA.

Of 78 respondents, 18 specified what they disliked about the videos. Responses were “It reminded me of myself/my child/someone I know who has got JIA” (7/18, 39%), “It made me feel emotional” (5/18, 28%), “The way it talks about JIA” (4/18, 22%), “It was cute” (2/18, 11%), and “It was funny” (2/18, 11%). One respondent expressed concern about self-protection, vulnerability, and inviting negative attention or bullying after sharing. Two respondents specified groups they wouldn't share the video with (school friends and work colleagues) stating it would make them feel vulnerable.

In terms of content, Video 1 was considered more appealing and shareable. One respondent commented that the tone of Video 2 was “gray and depressing;” another commented that the actor did not come across as “cool” and therefore the video might not be helpful in raising awareness and engagement with the condition and that teenagers might not want to be associated with an older person’s disease.

#### Telephone Follow-Up Interview Feedback

We were able to contact 15 participants for telephone feedback. Those contacted expressed support for the study and were positive and enthused about the video they had watched. Most felt that it was a good way to “get awareness out there.” One mother described the video (Video 2) as “quite catching” and said she wished she had seen it “2-3 years ago” when she first suspected that her daughter might have a medical condition. Participants tended to share the video with friends and family (either by social media—predominantly Facebook—or directly by email). They reported receiving positive feedback about the videos as well as consistent comments like “I didn’t realize that kids could get arthritis.” Telephone interviews further suggested that in most cases it was the parents or guardians who shared the link via their networks, mostly with family and friends but also through JIA-specific networks such as support groups and general parenting forums. Only 1 participant mentioned explicitly that her 13-year-old daughter shared the link with family and friends on Facebook and Instagram.

Two participants had not viewed the video when contacted and were prompted to do so. Reasons for not viewing included loss of the recruitment card, having to attend to family or health-related matters, and stress following their child’s recent diagnosis.

The video was also shared offline. One 13-year-old female shared the video with her teacher who then integrated it in a school lesson, although it was not widely shared online. The mother of a 9-year-old newly diagnosed female was very enthusiastic about the project but had recently stopped using social media. She suggested sharing the link through real-life networks and platforms including school notice boards and at extracurricular events.

## Discussion

### Principal Findings

This study has investigated the feasibility of seeding an online awareness campaign for JIA in a clinical setting and has demonstrated that this approach can work in what we believe is a unique study. We have shown that this approach is acceptable to most children and young people and their parents or guardians and that it can reach an audience which far exceeds the geographical and sociodemographic spread of other (eg, paper-based) information. Dissemination is influenced by the content and its shareability, participant engagement in social networks, and willingness to share personal or family-related health information.

The clinical motivation behind this study was to improve the diagnostic experience of patients with JIA. Our impression that awareness of JIA is low was supported by the initial group work in which children and young people and parents or guardians agreed there was little awareness of JIA in the community, schools, and primary care. Studies of delayed cancer diagnosis describe complex and varied pathways to diagnosis: delays can be patient-, family-, or doctor-related and occur between primary and secondary care or within secondary care [14]. Raising awareness in the community and within health care settings might address some of these areas and is one of the few tools available. However, although many campaigns have set out to do so [2,15,16], it is recognized that they may be ineffective or alternatively lead to increased and inappropriate demands on services [15].

Although this was a relatively small study, it demonstrated proof of concept for this technique; each link received a mean of 89.6 pageviews, and links were viewed from a wide range of geographic locations. Concentrations of views in the North East and London in the United Kingdom and within the United States likely reflect the connections of sharers locally as well as the population density and Internet usage in London and the United States.

Despite initial apparent enthusiasm from participants, links were not accessed by everyone recruited for the study. Unfortunately, we could not contact all of those concerned to understand why. The success of the project relied on a small number of enthusiastic sharers with appropriate viral dynamics or digital capital and access to and engagement with social media, factors which are crucial for successful seeding [17].

Despite the apparent social media engagement of younger digitally native patients, the most effective sharers were the parents or guardians (particularly mothers) of younger children, as predicted in the first phase. Although we had limited feedback from this population, likely factors for disengagement of JIA patients include unappealing tone and content of videos, reservations about sharing personal health-related information online, and lack of interest in the research. Other approaches like encouraging participants to make their own videos or sharing offline might be more successful.

A personal or emotional attachment to the message is important for sharing; the majority shared the video because their child or someone they knew had JIA. The engagement of patients and parents or guardians in spreading a health message represents a reconfiguration of the roles of patients and health professionals in a process which may require monitoring by public health professionals [18]. When spreading a health message online, a distinction between spreading information and awareness may be useful. We focused on the latter and tried to keep the message as simple as possible, reasoning that viewers could use the associated links to access further information. This approach, however, may reduce the effectiveness of educating the wider community.

In terms of absolute numbers of pageviews, Video 1 was more successful than Video 2 (2868 vs 368 views). However, this appeared to be driven by a couple of very successful sharers of Video 1 and not because of a significant difference in the number of pageviews. This emphasizes the impact of small numbers of highly connected and motivated individuals in online sharing. Comments about the videos appeared to favor the lighter and more humorous tone of Video 1. The online content which is shared to some extent reflects on the sharer, and it may be that sharers were happier to be associated with this than the darker tone of Video 2. Facebook was the dominant social network through which links were shared, predominantly to friends or family members. Most views were on mobile or tablet platforms.

Key to the transferability of this approach is the condition in question, and this approach may be more applicable to some conditions than others. Given the reservations about what sharers thought the messages said about themselves and their condition, messages about different conditions (eg, cancer or sexual health) are likely to be shared differently. Therefore, while there are clear elements of transferability, relevant condition-specific enablers and barriers should be taken into account when developing Web-based health messages.

### Limitations

A major drawback to our study is that although we had viewing statistics, we were unable to measure whether our method had raised awareness of JIA in the population or among those who watched the videos. Testing this concept within a formal study to which patients were required to consent led to some logistical difficulties including the uneven randomization of patients across the 2 videos. Our method of sharing written links to the videos was not ideal; future studies should consider direct electronic sharing of links (either by email, text message, or QR code) to eliminate the need to manually enter the address into a browser and facilitate the sending of reminders if appropriate. We believed that up to 6 months after diagnosis patients would be more motivated to share videos but were unable to test this. Our study was heavily dependent on 2 professionally produced videos, which were relatively expensive to produce and may limit the scalability and transferability of this approach. Furthermore, the dependence on the video content itself means it is possible that different videos would have generated entirely different results. The feedback we received was predominantly from women aged 25 to 44 years, which may not reflect the majority of the viewing audience.

In developing an effective awareness campaign, it might be appropriate to target other groups (eg, health professionals). We could not tell to what extent the links had been shared with health professionals, and some research into sharing behavior might be appropriate. While awareness campaigns can improve outcomes for patients, the impact on primary and secondary care of increased numbers of referrals as a result of a campaign should be considered [19].

### Conclusion

The findings of this exploratory study suggest that distributing a link to a shareable online video in a clinical setting is a feasible and potentially effective way of spreading a health message. The tone and content of the message are important factors in the success of this approach, as is an understanding of the population (patients and parents or guardians) most likely to share the resource. Other factors include the condition itself, the willingness of sharers to be identified with the condition, and preexisting awareness of the condition. The parents or guardians of affected children may be the most effective group for spreading health messages about childhood-onset conditions, and future campaigns should consider this. Further work should focus on refining this approach, delivering it at lower cost, and improving its generalizability across age groups and medical conditions.

## Appendix

Multimedia Appendix 1 Video 1. How old do you need to be . . .?

Multimedia Appendix 2 Video 2. Young to Old.
